# What Is the Relationship of Medical Humanitarian Organisations with Mining and Other Extractive Industries?

**DOI:** 10.1371/journal.pmed.1001302

**Published:** 2012-08-28

**Authors:** Philippe Calain

**Affiliations:** Unité de Recherche sur les Enjeux et Pratiques Humanitaires (UREPH), Médecins Sans Frontières, Genève, Switzerland

## Abstract

Philippe Calain discusses the health and environmental hazards of extractive industries like mining and explores the tensions that arise when medical humanitarian organizations are called to intervene in emergencies involving the extractive sector.

**Summary Points**

- In developing countries, extractive industries (including ore mineral mining and oil extraction) have far reaching consequences on health through environmental pollution, some communicable diseases, violence, destitution, and compromised food security.
- The rapid expansion of extractive industries and the increasing frequency of environmental disasters are bound to engage medical humanitarian organisations in developing novel types of expertise.
- While humanitarian organisations might be called to intervene in areas occupied by the extractive sector, in this Essay I argue that oil and mineral exploitation reveals a fundamental clash of values between humanitarianism, the for-profit sector, and privatised global philanthropy.
- Specific medical humanitarian organisations can respond to these challenges in different ways, based on their position between pragmatic or principled approaches, and their willingness to develop new technical capacities.

Since 2010, Zamfara State in northern Nigeria has been the theatre of what some reports have called “the worst lead poisoning epidemic in modern history” [1]. At least 43 villages have been affected during the outbreak period [2] and more than 400 children have died since March 2010 [3]. Contamination has been the result of small-scale artisanal mining and domestic processing of lead-heavy gold ore. An epidemiological survey conducted in May 2010 in two of the most affected villages [4] recorded a daily under-5 mortality rate (U5MR) more than six times the United Nations High Commissioner for Refugees (UNHCR) emergency benchmark of >2.0/10,000/day, with those aged less than 24 months being particularly at risk. The survey concluded that 25% of children aged <5 years had died in the past year and blood sampling indicated that most of the surviving children had critical blood lead levels that required chelation therapy. The long-term neurological and cognitive effects among survivors of this outbreak are still unknown.

A protracted public health crisis of such a magnitude, affecting predominantly young children in impoverished communities, has much in common with classical humanitarian crises created by famine, natural disasters, infectious diseases, or conflicts. However, acute lead poisoning is a medical condition about which team members of Médecins Sans Frontières (MSF) were not exactly familiar when they were initially called to investigate the Zamfara outbreak in March 2010. The organisation responded to this unusual situation by developing a novel field of expertise, notably in providing clinical care to the many children in need of chelation therapy [5]. MSF's continuing intervention in Zamfara is only part of a broader multisectoral public health intervention, including environmental remediation and the institution of safer mining techniques [6].

This context is not foreign to the usual terrain of medical humanitarianism, which combines clinical care, public health interventions, and advocacy for vulnerable populations. But at the same time, the Zamfara outbreak was a landmark in MSF's history, expanding the scope of medical humanitarian action to environmental emergencies. Indeed, the rapid expansion of mainstream development agendas will inevitably lead many relief organisations to confront the longer-term consequences of “extractive industries,” as it is becoming more and more known how chronic the adverse effects of such industries can have on health and societies. In this Essay I describe the broader health effects of extractive industries, to which relief organisations like MSF could be called to respond. In the last two sections, I will reflect upon the risks for these organisations in becoming engaged with corporate extractive industries.

## Extractive Industries and the Resource Curse

Extractive industries, or mining, deal with two categories of resources: (i) fossil fuels (coal, oil, or gas), and (ii) mineral ore. The exploitation of these resources can take variable dimensions, from large mining sites (generally run by multinational companies) to small-scale artisanal mining (as in the case of Zamfara). Unfortunately, for the majority of populations living in areas occupied by extractive industries destitution is often the norm, rather than the improved livelihoods that many hope would result from increased resource activity. This phenomenon has been variably called the “paradox of plenty” or the “resource curse.” It is typically, but not exclusively, observed in sub-Saharan Africa, where considerable reserves of oil and mineral resources are increasingly being exploited. The resource curse was initially defined as the apparent macro-economic observation that dependence on exports of natural resources slows down economic growth. Exact mechanisms are still disputed, but resource curse theories have been expanded to include broader observations than just macro-economic trends (Box 1).

Box 1. Common Features of Environments Occupied by Extractive Industries and Typically Subject to a “Resource Curse” Phenomenon*

**Original Definition**

- Slow macro-economic growth

**Classical Explanatory Observations (Macro Level)**

- Crowding out of productive economic sectors (agriculture and manufacturing)
- Corruption
- Authoritarian regimes
- Civil war
- Lower spending on health care and education

**Additional Observations (Micro Level)**

- Environmental destruction, including decreased access to clean water
- Dysfunctional and inequitable health systems
- Higher child mortality
- Higher rates of chronic malnutrition
- Lower life expectancy
- Violence and related health effects

* Adapted from reference [8].

The Niger Delta in Nigeria is a classical example of a resource curse environment based on oil extraction [7], but most oil-rich areas of sub-Saharan countries also feature unmistakable elements of a resource curse, for example southern Chad and South Sudan [8]. In a similar way, with their important deposits of gold and other valuable metals, the provinces of eastern Democratic Republic of Congo (DRC) have been held as a paradigm of destitution and transnational exploitation in the face of mineral wealth [9]. The resource curse also concerns countries outside of sub-Saharan Africa, including Mongolia, Timor-Leste, and Papua New Guinea.

Aside from macro-economic considerations, the enormous social, health, and environmental costs of extractive industries need to be put in the perspective of any real or alleged gains. Extractive resources are limited by definition. There is some consensus that the peak of world oil production is inevitable, if not yet past [10]. Over the last century, the global extraction of ores and minerals has increased by a factor of 27 [11]. This is not only due to the expansion of the world population, but also to an increase of average resource use per capita, mostly in industrialised countries. Even by the most conservative estimates, this situation is unsustainable. Furthermore, extracted resources should be seen through the lens of their genuine use value, instead of their exchange value or symbolic fallacies attached to it. For example, it is relevant to ask if the possession or consumption of an extra ounce of gold or another carat of gems is worth the human price exacted at their sites of exploitation. Humanitarian considerations can offer some illuminating perspectives on such questions.

## Health Hazards

Mining in general, and resource curse environments in particular, have been a source of increased health hazards for resident populations and workers alike. To the extent that safer environments, better education, better access to health care, and decreased poverty are important determinants of health, it is easy to see possible links between extractive industries, the resource curse, and poor health. In addition, a number of specific medical conditions have been documented as resulting from the activities of extractive industries, including oil exploitation.

For example, occupational and environmental hazards include exposure to pollution generated by mining and ore processing, pulmonary diseases due to dust inhalation (silicosis), respiratory diseases and cancer linked to gas flaring [7], and a number of communicable diseases such as tuberculosis and HIV/AIDS. The Blacksmith Institute, an international environmental nongovernmental organisation (NGO), lists mercury and lead pollution from mining or ore processing among the top ten worst toxic pollution problems in the world [12], and ranks as number one mercury pollution from artisanal gold mining. Mineral mining in southern Africa has been shown to be associated with extremely high incidence rates of tuberculosis [13], and in the same context, migration to and from mines contributes to HIV and tuberculosis spread in the general population. Mining towns also feature complex social environments open to risky sexual behaviours, with the resulting increase in sexually transmitted infections [14],[15]. More sporadic diseases can result from artisanal gold mining, like the protracted outbreak of Marburg hemorrhagic fever of Durba in northeastern DRC [16] that was apparently due to exposure to cave-dwelling bats [17]. The list of specific hazards also includes exposure to radionuclide mining and extraction. For example, concerns are being voiced over environmental contamination of soil, drinking water, inhaled dust, and urban constructions around uranium mines in northern Niger [18].

More general effects of extractive industries are likely to occur through impacts on arable land and water resources [19]. In DRC, chronic childhood malnutrition (stunting) is unequally distributed geographically between provinces. Rates of stunting remain very high in the Kasai and Katanga regions that rely on the mining industry, comparable to the level seen in eastern provinces that are at war [20]. Mining thus appears to be an independent social determinant of chronic malnutrition in DRC.

Finally, oil and mineral extraction often generate contexts of chronic violence, civil war, and human rights abuses, which are classical features of the resource curse. Mental health consequences of chronic violence and insecurity in the Niger Delta have been documented, including a high prevalence of post-traumatic stress disorder [21]. In eastern DRC, a complex pattern of socio-cultural disarray generates different forms of violence [9], and currently there is an endemic situation of “rape with extreme violence” across mining zones in South Kivu [22].

## Differing Values between Humanitarian Organisations and Extractive Industries

The overview presented above shows that there is much scope for medical humanitarian organisations, through their logistic resources, technical expertise, and means of advocacy, to help improve the health situation of populations impacted by extractive industries. This can be the case, for example, in response to acute environmental emergencies. Humanitarian actions can also offer temporary contributions to health care provision in endemic contexts of violence and destitution. With the consumption of oil and minerals increasing worldwide, humanitarian organisations will very likely be expected to play an even more important and frequent role in the mitigation of the health impacts of extractive industries. By doing so, however, humanitarian organisations will inevitably be brought to confront the root causes and mechanisms of such adverse impacts, and the conflicts of values that they represent. Three examples come to mind: the globalisation of economic forces, corporate governance, and global philanthropy.

Firstly, with regard to the informal sort of economy grounded in artisanal or illegal mining, it is important to examine the deeper social and political causes of destitution that lead to a shift in local livelihoods from agriculture to mineral extraction. For example, the recent gold rush in Zamfara and its dire public health consequences ultimately reflect a volatile global economy artificially driving the value of gold [23]. Secondly, in the case of extractive industries controlled by the more formal corporate sector, it is important to recognise the clash of values between humanitarian action and for-profit industries. For example, the presence of medical NGOs could be misconceived as contributing to the “social license to operate” of extractive industries, particularly when paralleled by local corporate initiatives undertaken under motives of “corporate social responsibility” [8]. In addition, humanitarian NGOs can be directly engaged as actors of privatisation and outsourcing of health services, particularly in conflict settings [24], again representing a conflict of values. Thirdly, at a higher level of management, NGOs can become directly or indirectly the beneficiaries of philanthropy exercised by private foundations with financial interests in the extractive sector. Stuckler and colleagues [25] have shown how the financing of global health philanthropy is prone to conflict of interests, through investments in corporate sectors that can contribute to adverse effects on health and social welfare. In 2010, for example, the Bill & Melinda Gates Foundation had more than 5% of stock investments in transnational oil companies (Text S4 in [25]), some of them operating in the Niger Delta. From the Foundation's perspective, the separation between grant-making and investing functions [26] accommodates such a contradiction, but from the perspective of local populations, this could be seen as an example of disregard for the root causes of their ill health brought about by mainstream development programs relying on the rapid extraction of natural resources.

## A New Agenda for Medical Humanitarian Organisations?

The health community has been accused of failing to do its share to address the kind of social injustice epitomised by the condition of residents of the Niger Delta [27]. As they witness resource curse contexts and contribute to some local relief, medical humanitarian organisations will inevitably be called forth to add their voice to broader debates on the adverse health consequences of extractive industries. In case of environmental emergencies, this moral imperative could be fulfilled by advocating for more fundamental and collective remediation measures. For example, MSF recently lambasted Nigerian authorities for delaying the urgently needed release of funds earmarked for environmental remediation and safer mining [28]. While advocacy and expansion of their operational expertise are obvious paths for NGOs, a more complex and ideologically loaded question to solve is about what type of relationships humanitarian organisations should entertain with the corporate sector. At the institutional level, three options can be envisaged.

Firstly, some NGOs enter into *broad alliances* with corporate industries, following expectations of mutual benefits, shared expertise, and a space for dialogue. For example, the Devonshire Initiative [29] was established under the auspices of the University of Ottawa and brings together prominent international humanitarian NGOs, Canadian mining companies, and Canadian development agencies as observers. Such alliances have been criticised as being improperly imposed upon NGOs by conservative governments, through conditional funding by their respective official development agencies [30]. Secondly, some NGOs make *conditional alliances* with the corporate sector, based on common norms or endeavours, for example the respect of human rights or fair trade. Examples include the Kimberley Process [31] and the Alliance for Responsible Mining [32]. Thirdly is *lack of engagement* with the corporate sector, which many mainstream medical humanitarian organisations would rather opt for due to questions of values and conflicts of interest. But there are additional operational reasons why medical humanitarianism may wish to remain independent from the corporate sector. In the frequent cases where resource curse situations fuel civil unrest, extractive industries are naturally perceived as parties in the conflict. A broad interpretation of the main Red Cross principles (neutrality, impartiality, and independence) would therefore suggest that organisations ascribing themselves to the Red Cross tradition ought to interact carefully with the corporate extractive sector, in the same way as they would avoid partnerships with military forces. More generally, a principled understanding of humanitarian medicine entails selfless moral commitments that are incompatible with the for-profit objectives of corporate industries. Accordingly, a pragmatic approach of engagement with the corporate sector for the delivery of aid, or an implicit support to mainstream development agendas could compromise the legitimacy of humanitarian medicine [33], particularly in the eyes of those populations suffering the most from the presence of extractive industries.

A fourth and additional line of engagement would be for NGOs to *support the public sector* in its specific endeavours to address the health effects of extractive industries. Along this line, a recent initiative is the declaration of political commitment to fight tuberculosis in the mining sector, soon to be ratified by the Southern African Development Community (SADC), a regional inter-governmental organisation [34].

With an essentially private, broad-based, and sustained funding flow, MSF has not yet been compelled to take any position over its relationship toward extractive industries, but it is currently opening an internal debate on pragmatist versus principled arguments. In the meantime, the Zamfara outbreak has called attention to a novel agenda for medical humanitarian organisations, including technical preparedness for environmental disasters, dialogue with international environmental organisations, and a better understanding of the exact role of resources extraction in perpetuating humanitarian crises.

## Abbreviations

DRC: Democratic Republic of the Congo
MSF: Médecins Sans Frontières
NGO: nongovernmental organisation
SADC: Southern African Development Community
U5MR: under-5 mortality rate
UNHCR: United Nations High Commissioner for Refugees
